# Novel Surface Acoustic Wave Temperature–Strain Sensor Based on LiNbO_3_ for Structural Health Monitoring

**DOI:** 10.3390/mi13060912

**Published:** 2022-06-09

**Authors:** Xiangrong Li, Qiulin Tan, Li Qin, Xiawen Yan, Xiaorui Liang

**Affiliations:** 1 State Key Laboratory of Dynamic Measurement Technology, North University of China, Taiyuan 030051, China; 20050907@nuc.edu.cn (X.L.); qinli@nuc.edu.cn (L.Q.); yanxw603@163.com (X.Y.); wangshaner_0529@163.com (X.L.); 2 Science and Technology on Electronic Test and Measurement Laboratory, North University of China, Taiyuan 030051, China; 3 College of Science, North University of China, Taiyuan 030051, China

**Keywords:** surface acoustic wave, temperature sensor, strain sensor, wireless passive sensor

## Abstract

In this paper, we present the design of an integrated temperature and strain dual-parameter sensor based on surface acoustic waves (SAWs). First, the COMSOL Multiphysics simulation software is used to determine separate frequencies for multiple sensors to avoid interference from their frequency offsets caused by external physical quantity changes. The sensor consists of two parts, a temperature-sensitive unit and strain-sensitive unit, with frequencies of 94.97 MHz and 90.05 MHz, respectively. We use standard photolithography and ion beam etching technology to fabricate the SAW temperature–strain dual-parameter sensor. The sensing performance is tested in the ranges 0–250 °C and 0–700 μԑ. The temperature sensor monitors the ambient temperature in real time, and the strain sensor detects both strain and temperature. By testing the response of the strain sensor at different temperatures, the strain and temperature are decoupled through the polynomial fitting of the intercept and slope. The relationship between the strain and the frequency of the strain-sensitive unit is linear, the linear correlation is 0.98842, and the sensitivity is 100 Hz/μԑ at room temperature in the range of 0–700 μԑ. The relationship between the temperature and the frequency of the temperature-sensitive unit is linear, the linearity of the fitting curve is 0.99716, and the sensitivity is 7.62 kHz/°C in the range of 25–250 °C. This sensor has potential for use in closed environments such as natural gas or oil pipelines.

## 1. Introduction

Temperature–strain sensors are widely needed in quality, safety, and reliability monitoring in aerospace, automotive, urban piping, and civil engineering [1,2]. In these applications, the sensor must have high sensitivity, a wide detection range, and robust construction. Moreover, in closed or rotating environments, external connected antennas required by traditional wired sensors are difficult to connect; hence, wireless passive sensors are needed [3]. The wireless passive detection sensors include the inductor and capacitance (LC) sensor [4,5], microwave sensor [6,7,8], and surface acoustic wave (SAW) sensor [9,10,11]. LC wireless passive sensors adopt near-field coupling technology, which enables them to rapidly read test data and perform energy coupling under harsh environments but has a short reading distance and low performance [12,13,14]. The LC sensor can realize the integration of multiple sensors for multi-parameter measurement, but the integrated sensor is usually relatively large. Microwave backscattering technology is robust to interference and has a long transmission distance, but the device is difficult to integrate and does not meet the requirements for simultaneous monitoring of multiple parameters in harsh environments [15].

Using the SAW sensing mechanism, many sensors can be built for sensing gas, magnetism, current, pressure, angular velocity, and other chemical or physical measurements [16,17,18,19,20,21]. An SAW sensor is small, highly sensitive, and easy to integrate, and it is widely used in the integration of multi-parameter sensors [22,23,24]. A large number of SAW strain sensors have been reported. For instance, Wang et al. reported a wireless passive strain sensor based on SAWs. The sensitivity of the sensor is 598 Hz/μԑ with high sensitivity and good temperature stability and linearity [25]. Li et al. reported a high temperature–strain sensor that can measure temperatures up to 250 °C. Three separate sensors were used to decouple the strain sensor [26]. Chen et al. proposed a strain sensor using a dual-channel SAW delay-line oscillator with a strain sensitivity of 126 Hz/μԑ and a measuring range of 0–400 °C [27]. Donohoe designed and developed a special-purpose calibration apparatus and calibrated a standard single-port SAW resonator (SAWR) with an unbiased resonant frequency of 433.42 MHz. The response of this SAWR to longitudinal and transverse strain has been studied in addition to the strain sensitivity of SAWR to strain applied in different directions [28]. Stoney proposed an SAWR strain sensor that can be used for wireless passive differential strain measurement. Its temperature measurement range is from 20 °C to 100 °C, its strain measurement range is from −400 to 400 μԑ, and its maximum hysteresis is 0.49%, which can compensate for temperature and improve transverse axis sensitivity [29]. Anin et al. reported an LGS high temperature–strain sensor with a temperature test range of 25–400 °C and a strain sensitivity of 118 Hz/με at room temperature, which gradually decreases with the increase in temperature [30]. The above SAW strain sensor has good linearity in the measurement range; however, it can only measure a single parameter. Often, the measurement environment is complex: the temperature, humidity, and strain change simultaneously. In a complex environment, it is obvious that a single-parameter sensor cannot test multiple parameters at the same time. In addition, in high-temperature or temperature test environments, the output of strain measurement is the result of the combined measurement of temperature and strain. Strain tests are inaccurate because it is generally very difficult to extract the value of strain with a single sensor. The above SAW strain sensor did not achieve accurate strain in high-temperature environments. The integration of temperature and strain sensor may not only realize the measurement of temperature and strain but also facilitate the extraction of strain.

In this paper, we propose the integration of two parameters: temperature and strain. The frequencies of multiple sensors were determined using a COMSOL Multiphysics simulation, allowing the mutual interference of the frequency offsets of the multiple sensors caused by changes in external measurement physical quantities to be avoided. We fabricated a SAW temperature–strain dual-parameter sensor using standard lithography and ion beam etching. Lastly, the sensing performance was tested. The temperature sensor realized the real-time monitoring of the ambient temperature; the strain sensor detected the strain and temperature; and, together, they realized the decoupling of the strain and temperature through calculation.

## 2. Materials and Methods

### 2.1. Simulation Model of the Sensor

To design the temperature–strain dual-parameter sensor, simulations were first performed. First, the resonant frequencies of the two sensing units were determined using COMSOL Multiphysics simulation. Then, by applying the temperature field, the resonant frequency offset caused by changes in temperature were calculated. As long as the frequency difference between the two sensing units is greater than the frequency offset caused by changes in the environment, frequency crosstalk is not a problem. A two-dimensional simulation was also performed. Lithium niobate was selected as the substrate, and the tangential direction was 128° Y–X. To simulate the characteristic frequency, a simplified sensor model was used. There are many pairs of interdigital transducers and reflection gratings that produce SAWs, and they are also periodic, so a pair of interdigital electrode and mechanical and electrical periodic boundary conditions were used in the simulation. This not only reduces the amount of calculation but also maintains the authenticity of the wave propagation. In the simulation, the interdigital transducer adopts a uniform interdigital electrode structure; the widths of the fingers of the two sensing units are 9.5 μm and 10 μm, respectively; and the wavelengths are 38 μm and 40 μm, respectively.

**Figure 1:**
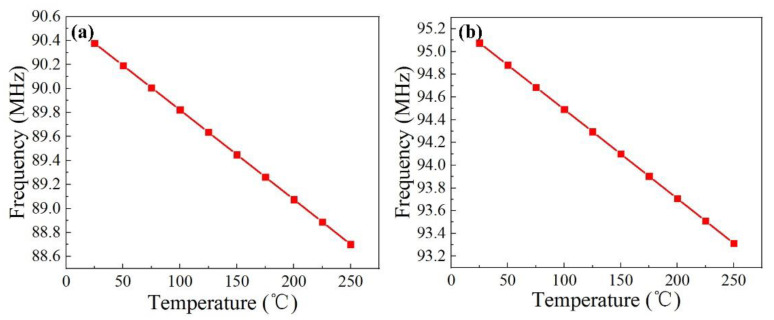
Simulation results of sensor temperature. (**a**) Response of temperature sensor to temperature. (**b**) Response of strain sensor to temperature.

The simulation obtained frequencies of 95.076 MHz and 90.3775 MHz, respectively, resulting in a frequency difference of 4.6985 MHz, which can realize the separation of the two frequencies. Figure 1 shows the relationship between the frequency and temperature of the temperature–strain sensing unit within the temperature range of 25–250 °C. The change in frequency of the temperature-sensitive unit is 1.6765 MHz (Figure 1a), and the change in frequency of the strain-sensitive unit is 1.763 MHz (Figure 1b). This design of the interdigital width of the sensing unit can avoid frequency crosstalk caused by temperature changes.

### 2.2. Strain Test Platform

**Figure 2:**
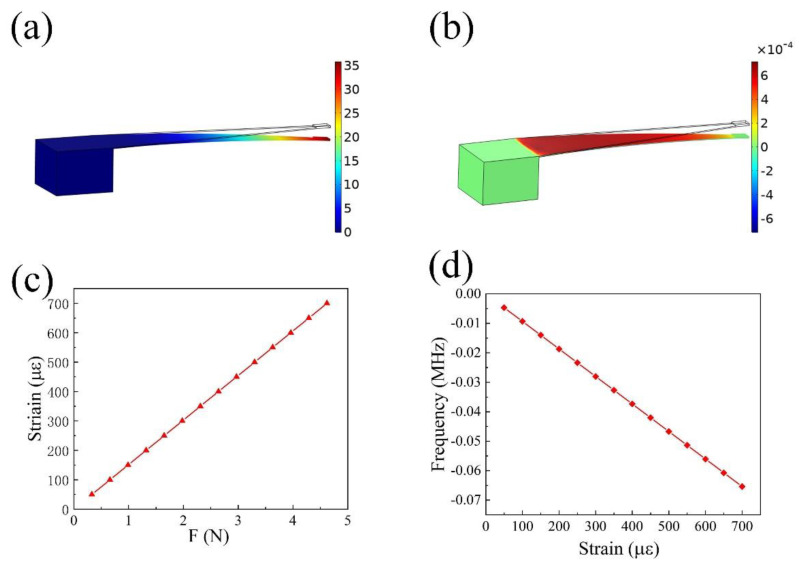
The strain test platform was simulated and designed. (**a**,**b**) Strain and displacement distribution of cantilever beam under 4.62 N pressure. (**c**) The relationship between the pressure loaded on the cantilever beam and the strain at the position of the strain sensor. (**d**) The relationship between the strain loaded on the sensor and the frequency shift of the strain sensor.

The strain test platform is the constant stress cantilever beam. The stress and strain of a constant stress cantilever beam were simulated using COMSOL Multiphysics with a simulation material of titanium–magnesium alloy material, as shown in Figure 2. When a load of 4.62 N is applied to the free end of the strain test platform, approximately uniform strain can be obtained near the fixed end of the cantilever beam on the X–Y plane. Moreover, the position of maximum strain is also close to the fixed end, and the strain near the free end is small, as shown in Figure 2b. The changes in strain under different stresses were simulated, as illustrated in Figure 2c, which shows that the strain increases with respect to the external force.

### 2.3. Sensing Mechanism

When the piezoelectric substrate is stressed and deformed, the width of the interdigital transducers of the sensor changes. When the substrate is stretched, the interdigital width increases, the wavelength increases, and the frequency decreases. Conversely, the wavelength decreases and the frequency increases when the substrate is compressed. The change in frequency is calculated by the following equation:

(1) $$\Delta f=-\frac{v\Delta \lambda }{\lambda^{2}}=-\;f\frac{\Delta \lambda }{\lambda }=-\;f\varepsilon_{x}$$

The frequency is proportional to the strain, and the change in frequency can hence be calculated from the strain.

The change in the width of the interdigital electrode caused by strain is not the only factor of frequency shift. Strain can also cause changes in the stiffness matrix and density of the base material, which leads to changes in the wave speed and frequency shift [31]. In this paper, the propagation direction of the surface acoustic wave is parallel to the strain direction. Therefore, the deviation in frequency caused by the change in wave velocity is small and can be ignored [32]. We only need to consider the frequency shift caused by the strain-induced change in the width of the interdigital electrode. Further, the frequency shift can be calculated from the simulated strain variables, as shown in Figure 2d. When the strain is 700 μԑ, the frequency change is 0.066 MHz.

As a result of the simulation, the widths of the interdigital electrode of the temperature-sensitive and strain-sensitive units of the proposed dual-parameter sensor were determined to be 9.5 μm and 10 μm with wavelengths of 38 μm and 40 μm, respectively. The two frequencies are separated, and the interference between frequency offsets caused by temperature and strain changes can be avoided.

When the temperature changes, the width of the interdigital transducers of a sensor change due to thermal expansion, resulting in a change of wavelength. Changes in temperature also change the density, elastic coefficient, piezoelectric coefficient, and dielectric constant of the substrate, resulting in changes in the wave velocity of the acoustic surface. Changes in the wavelength and speed of an SAW cause changes in frequency, and the frequency change can be calculated by the following formula:

(2) $$\Delta f=f\frac{dv}{v}-\;f\frac{d\lambda }{\lambda }$$

Hence, the temperature of the environment can be detected by measuring changes in the frequency of the sensor.

### 2.4. Fabrication of the Sensor

The SAW temperature–strain sensor is composed of two parts, a temperature-sensitive unit and a strain-sensitive unit. Each sensitive unit adopts a single-port resonator, consisting of interdigital transducers and a pair of reflective gratings. The interdigital transducers are composed of 100 pairs of equal-interval fingers, and the reflective grating is composed of 150 pairs of short-circuited reflectors. As noted above, the strain-sensitive unit has a width of the interdigital electrode of 9.5 μm and a wavelength of 38 μm. The temperature-sensitive unit has a width of the interdigital electrode of 10 μm and a wavelength of 40 μm. In the experiment, the substrate is lithium niobate, and the pattern was drawn using standard photolithography and ion beam etching.

**Figure 3:**
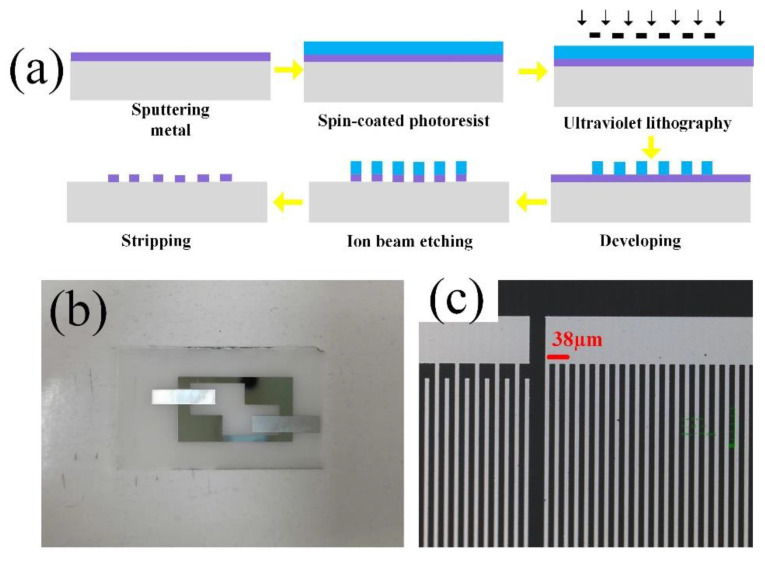
(**a**) The process of sensor preparation. (**b**) Optical image of the prepared sensor. (**c**) Confocal microscope image of the interdigital transducers structure.

Figure 3a shows the fabrication process of the device. First, the substrate was cleaned with acetone and alcohol, and then the metal layer Cr/Pt (20/180 nm) was sputtered on the substrate by magnetron sputtering. Cr was first sputtered to increase the adhesion between the substrate and metal. The pattern was then formed using a standard lithographic process. The metal outside the figure was etched using an ion beam. After the device was soaked in acetone for half an hour, ultrasonic stripping was performed. Figure 3b shows an optical image of the device, and Figure 3c presents a microscopic view of the interdigital electrode, which shows that the metallization rate is 0.5..

## 3. Results and Discussion

### 3.1. Test Platform

**Figure 4:**
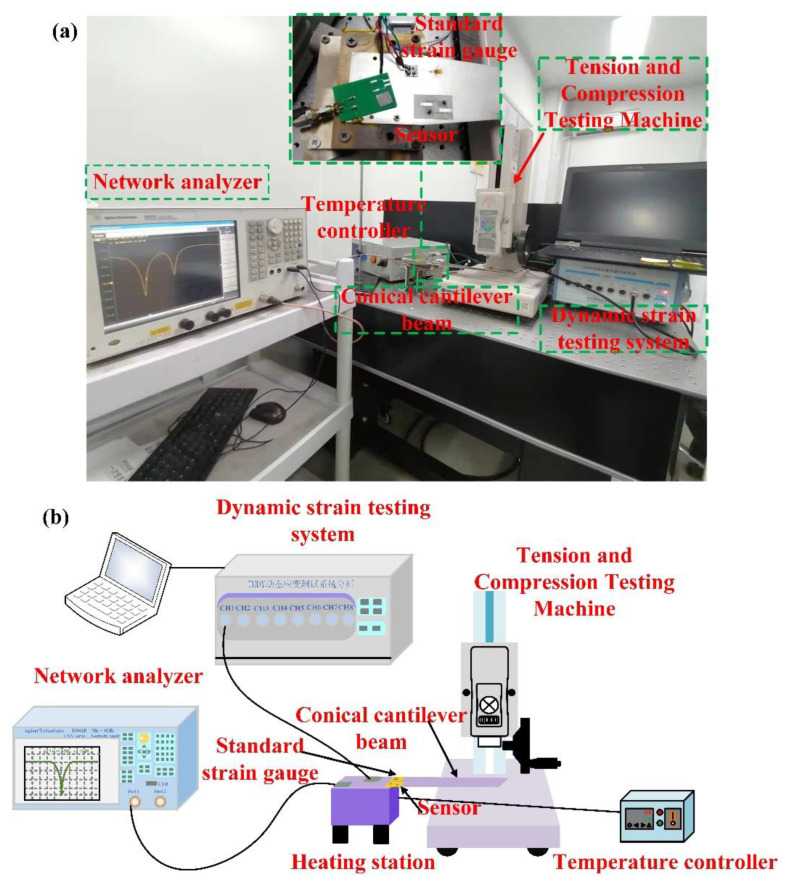
(**a**) The measurement platform for the measurement of temperature–strain parameters. (**b**) Schematic diagram of temperature–strain mixing test platform.

The temperature test device is composed of a temperature control box and a heating platform. The strain test platform is the constant stress cantilever beam designed by us. The strain platform is fixed on the heating platform using a clamp so that the sensor can measure the temperature and strain on the composite test platform. A network analyzer was used to test the frequency and frequency shift of the sensor unit as a function of temperature and strain. Using high-temperature glue, the strain sensor was pasted to the position of maximum strain. The temperature sensor was placed at the fixed end and was not pasted to the strain test platform. In this way, only the strain sensor can perceive the strain when an external force is loaded at the free end, whereas the temperature sensor is not affected by the strain and its frequency does not change. A standard strain gauge was pasted on a position symmetrical to the SAW strain sensor to calibrate it, as shown in Figure 4. The standard strain gauge was connected to a terminal, and the terminal was connected to the dynamic strain testing system through a cable. The strain testing system was connected to a computer, and the dynamic measuring system of strain gauge on the computer recorded the changes in the standard strain gauge. The solder pad of the sensor was connected with silver wire to the solder pad of the printed circuit board (PCB). The solder pad of the PCB was connected to a Sub Miniature version A (SMA) head. The SMA head and the network analyzer were connected by the cable wire.

### 3.2. Temperature and Strain Sensing Performance

**Figure 5:**
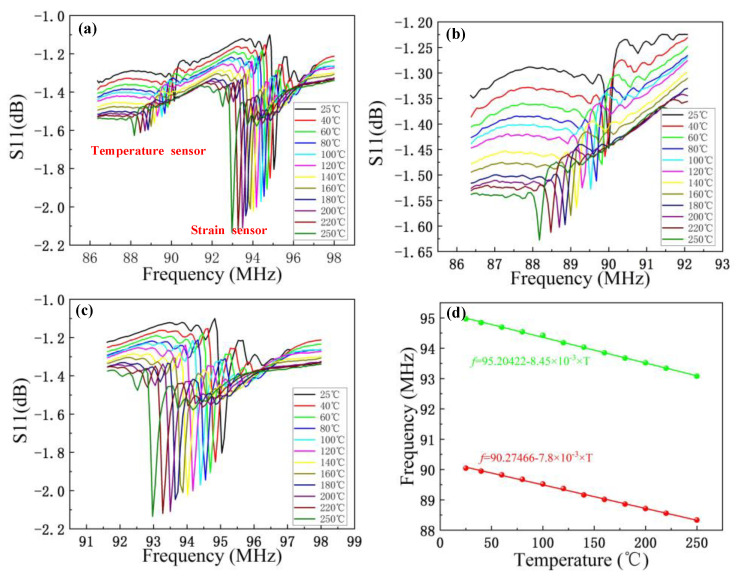
Temperature test results of the temperature–strain integrated sensor. (**a**) Temperature response curve of the dual-parameter sensor from 25 °C to 250 °C. (**b**) Enlargement of the temperature response curve of the temperature sensor. (**c**) Enlargement of the temperature response curve of the strain sensor. (**d**) The relation curve of temperature and frequency of each sensitive unit of the integrated multi-parameter sensor.

The temperature sensing abilities of the temperature and strain sensors were tested by measuring the S11 value of the temperature and strain sensors with respect to changes in temperature. The results are presented in Figure 5a, which shows that the frequencies of the temperature- and strain-sensing units decrease with increases in temperature. As the temperature increases, the speed of the SAW decreases. The widths of the fingers increase because of thermal expansion, resulting in longer wavelengths, so the resonant frequencies of the sensors decrease. Figure 5b,c are enlarged images of the results of the two sensors in Figure 5a, and they clearly show the variation in frequency with temperature. The frequency was further extracted from the S11 curve, and the relationship between frequency and temperature was fitted, as shown in Figure 5d. It can be seen from the figure that the temperature of the temperature-sensitive unit changes linearly with frequency. The linearity of the fitting curve is 0.99716. In the range of 25–250 °C, the change in frequency is 1.7134 MHz, and the sensitivity is 7.62 kHz/°C. The temperature of the strain-sensitive unit also varies linearly with frequency; the linearity of the fitting curve is 0.99705. In the range of 25–250 °C, the frequency variation is 1.8869 MHz, and the sensitivity is 8.38 kHz/°C. The results show that a higher frequency of the sensing unit leads to a higher sensitivity. There is a small deviation between the experiment and simulation, which is caused by the deviation between the simulated material and the actual material and the tolerance in the manufacturing process.

**Figure 6:**
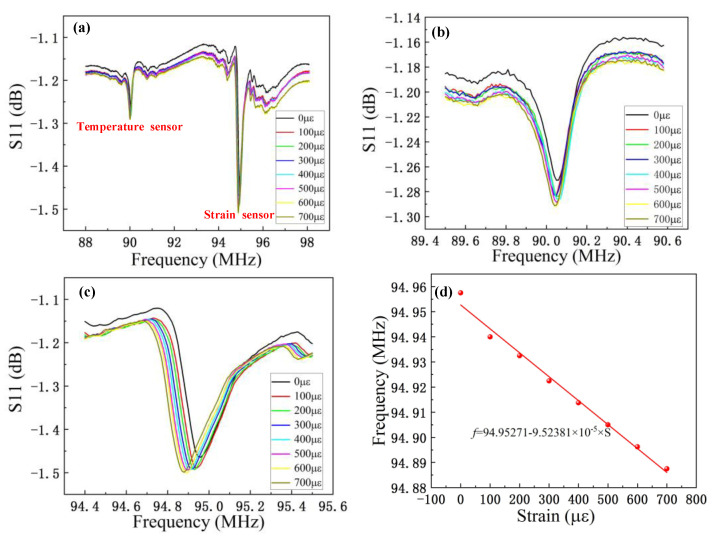
Integrated two-parameter sensor response to strain. (**a**) Strain response curve of the dual-parameter sensor at 25 °C for 0–700μԑ. (**b**) Enlargement of the strain response curve of the temperature sensor. (**c**) Enlargement of the strain response curve of the strain sensor. (**d**) Curve displaying the relationship between the strain and frequency of the strain-sensitive unit.

To test the strain-sensing ability of each sensing unit, we conducted further tests on the temperature and strain test platform. The strain sensitivity of each unit was tested in a temperature range of 25–250 °C and a strain range of 0–700 μԑ. We first tested the S11 curves under different strains at room temperature, as shown in Figure 6a. Figure 6b shows the response of the temperature-sensitive unit to strain. The strain varies within the range of 0–700 μԑ, but the frequency of the temperature-sensitive unit does not change when pressure is applied. This is because the temperature-sensitive unit is located at the fixed end of the platform. The size of its fingers does not change when external forces are applied to the free end of the platform. When a force is applied at the free end, the strain test platform deforms. Because the sensor is stuck to the strain platform, the strain of the test platform is transferred to the sensor, and the sensor also deforms, resulting in changes in the center frequency of the SAW strain sensor. Figure 6c presents the S11 curve of the strain-sensing unit, which shows that the frequency of the strain sensor decreases with increases in strain. The main reason is that when the strain increases, the width of the cross-finger electrode increases; that is, the wavelength increases, resulting in a decrease in frequency. Strain will also lead to changes in propagation velocity, but these changes are small, so the influence on frequency can be ignored [32]. Figure 6d shows the relationship between the frequency of the strain-sensitive unit and strain. The relationship between the strain and the frequency is linear. In the range of 0–700 μԑ, the variation in strain is 70 kHz, and the sensitivity is 100 Hz/μԑ.

**Figure 7:**
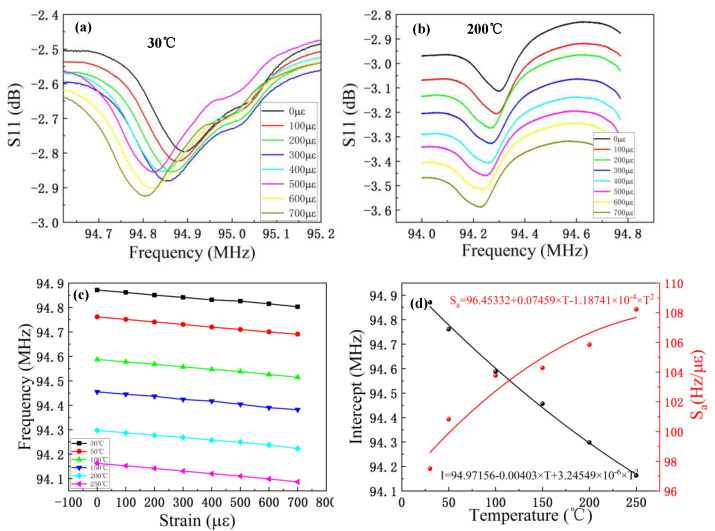
Strain test results of strain-sensing unit under different temperature environments. (**a**) Strain response curve of the strain sensor at 30 °C for 0–700 μԑ. (**b**) Strain response curve of the strain sensor at 200 °C for 0–700 μԑ. (**c**) The relationship between resonant frequency and strain of the strain-sensing unit under different strains at 30~250 °C. (**d**) Polynomial fitting of the slope and intercept of the curve of resonance frequency variation with strain at different temperatures.

We next tested the response of the strain sensor to strain at different temperatures. Figure 7a presents the relationship between the S11 value and the frequency of the strain sensor at 30 °C. The strain test range is 0–700 μԑ, and the resonant frequency decreases with increases in strain. Figure 7b presents the relationship between the S11 value and the frequency of the strain sensor at 200 °C. As above, the test range of the strain is 0–700 μԑ. The frequency was then extracted from the test results at different temperatures, and the variation in frequency with respect to strain at different temperatures was plotted, as shown in Figure 7c. As this figure shows, the relationship between the resonant frequency and strain is linear at different temperatures, and the frequency decreases with the increase in temperature, whereas the frequency decreases linearly with the strain at the same temperature.

In a complex environment, the temperature and strain change at the same time, and the output frequency signal is a result of both the temperature and strain. Therefore, the frequency change term is affected by both the temperature and strain, as shown in Figure 7c. In practical applications, accurate measurement of the strain at an arbitrary temperature is key. There is a linear relationship between strain and resonant frequency at a specific temperature, and each curve has a definite slope and intercept, as shown in Figure 7c. The intercept and slope of the fitting curve in Figure 7c were extracted under a strain range of 0–700 μԑ and temperature range of 30–250 °C, and the polynomial fitting curve of the intercept and slope is presented in Figure 7d. The fitting lines of each strain at any temperature can be expressed as follows:

(3) $$f_{s}(T)=\mathrm{incertept}(T)+S_{a}*S$$

**Figure 8:**
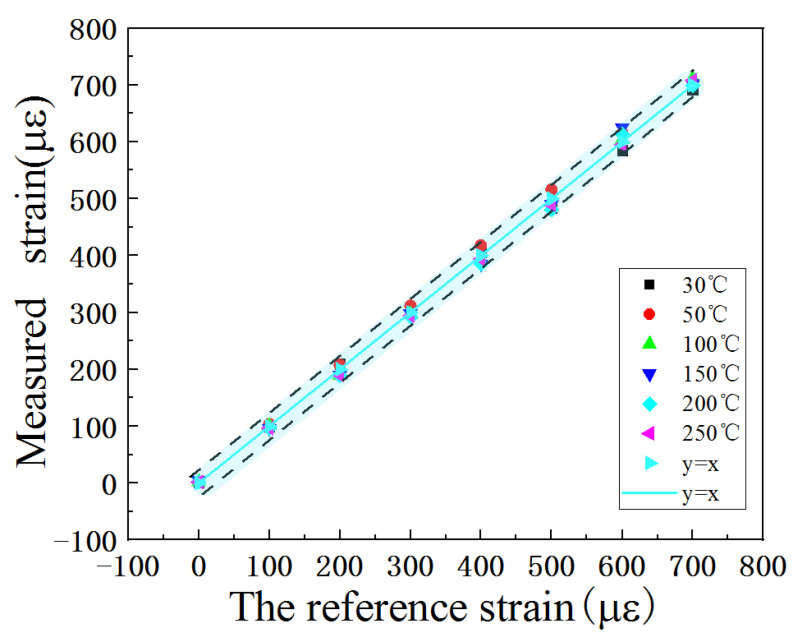
Error analysis of fitting results and test data.

The ambient temperature was tested by the temperature sensor, and the strain was calculated using Equation (3). The calculated error between the measured strain and the reference strain is shown in Figure 8, which is within the range of ±4.62%. The error may be caused by inaccurate measurement and the approximate linear fit shown in Figure 7c.

In addition, compared with other SAW-based strain sensors, our sensor can achieve the measurement of both temperature and strain parameters, as shown in Table 1.

## 4. Conclusions

We proposed a temperature–strain dual-parameter sensor based on SAWs that can detect temperature and strain simultaneously. The proposed sensor realizes temperature and strain frequency separation. The sensor was fabricated on a lithium niobate base using standard lithography techniques. Using a special mounting method, only one of the sensors can sense the strain, whereas the other sensor does not respond to the strain and only senses the temperature. The prepared dual-parameter sensor can operate stably in an environment of 0–700 μԑ and 25–250 °C. The frequency of the temperature-sensing unit changes by 1.7134 MHz in the range of 25–250 °C, and the sensitivity is 7.62 kHz/°C. Moreover, the sensitivity of the strain-sensing unit is 100 Hz/μԑ in the range of 0–700 μԑ at room temperature. The response of the strain-sensing unit to strain at different temperatures was tested, and the extracted values were fitted so that the strain values could be extracted from the results. This temperature–strain sensor enables wireless passive monitoring and has the potential to be used in enclosed environments. This sensor will be suitable for strain and temperature measurement in pipelines transporting natural gas or oil.

## Figures and Tables

**Table 1:** Comparison between the sensors we studied and previously reported sensors.

The Basal	Sensitivity (Hz/μԑ)	Range	Integration	Reference
LGS	142	25–250 °C/700 μԑ	No	[26]
128° Y-X LN	126	400 μԑ	No	[27]
36 (AT) quartz	479	400 μԑ	No	[28]
128° Y-X LN	100	25–250 °C/700 μԑ	Yes	This work

## Data Availability

Data sharing is not applicable to this article as no new data were created or analyzed in this study.
